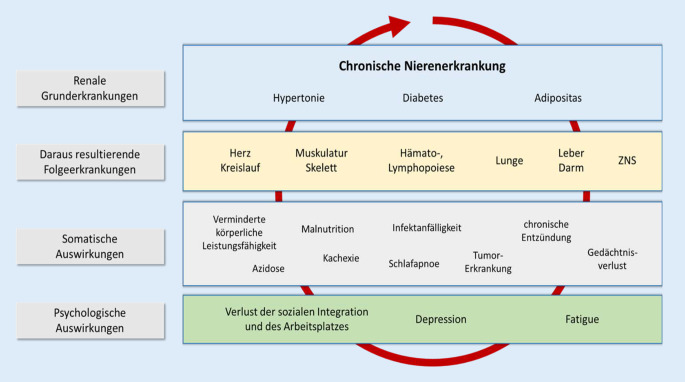# Langfristige körperliche und psychische Folgen chronischer Nierenerkrankungen

**DOI:** 10.1007/s00103-022-03515-0

**Published:** 2022-03-21

**Authors:** Friedrich Thaiss

**Affiliations:** 1 III. Medizinische Klinik und Poliklinik, Universitätsklinikum Hamburg-Eppendorf, Martinistr. 52, 20246 Hamburg, Deutschland; 2 BBraun, Viamedis, MVZ DTZ, Waldshut-Tiengen, Deutschland

**Keywords:** Chronische Niereninsuffizienz, Nephrologie, Psychonephrologie, Langzeitfolgen, Überleben, Chronic kidney disease, Nephrology, Psychonephrology, Long-term complications, Long-term survival

## Abstract

Aufgrund der verbesserten Behandlungsoptionen können Patient:innen mit chronischen Nierenerkrankungen heute deutlich länger überleben als noch vor 10 Jahren. Das Überleben ist für die Betroffenen jedoch immer mit einem Verlust an Lebensqualität verbunden. In diesem Beitrag wird eine kurze Übersicht über die körperlichen und psychischen Erkrankungsfolgen, Begleiterkrankungen und Therapienebenwirkungen bei chronischen Nierenerkrankungen gegeben. Auf bisher bekannte Auswirkungen der COVID-19-Pandemie wird hingewiesen. Abschließend wird aufgezeigt, wie die Langzeitbehandlung weiterentwickelt werden sollte, um die Lebensqualität der Patient:innen zu erhöhen.

Funktionseinschränkungen der Niere haben aufgrund der Kontamination des Blutes mit harnpflichtigen Substanzen (Urämie) schwere Auswirkungen auf den Gesamtorganismus. Zusätzlich sind die Patient:innen von Nebenwirkungen betroffen, die im Zusammenhang mit der medikamentösen Therapie, Dialyse oder Nierentransplantation auftreten können. Patient:innen und Angehörige sind einer großen psychischen Belastung ausgesetzt. Infektionen mit SARS-CoV‑2 können die Nierenfunktion beeinträchtigen und auch die Prognose einer bereits bestehenden Erkrankung verschlechtern.

Die ganzheitliche Versorgung der Patient:innen mit chronischen Nierenerkrankungen muss neben der medizinischen Versorgung auch die psychologischen und psychosozialen Aspekte berücksichtigen. Nephrologie und Psychonephrologie müssen Hand in Hand weiterentwickelt werden, um die medizinische Versorgung und Lebensqualität der betroffenen Patient:innen zu verbessern.

## Abstract (en)

Due to improved treatment options, patients with chronic kidney disease can survive significantly longer than even 10 years ago. However, survival is always associated with a loss of quality of life for those affected. This article provides a brief overview of the physical and psychological disease consequences, concomitant diseases, and therapy side effects. Reference is made to previously known effects of the COVID-19 pandemic. Finally, it will be shown how long-term treatment should be further developed in order to improve patient quality of life.

Functional impairment of the kidney has severe effects on the entire organism due to contamination of the blood with urophanic substances (uremia). In addition, patients are affected by side effects that can occur in connection with drug therapy, dialysis, or kidney transplantation. Patients and their relatives are exposed to great psychological stress. Also, infections with SARS-CoV‑2 can impair kidney function and worsen the prognosis of a pre-existing disease.

The holistic care of patients with chronic kidney disease must consider not only medical care but also psychological and psychosocial aspects. Nephrology and psychonephrology must be further developed hand-in-hand to improve the medical care and quality of life of affected patients.

## Einführung

Die Behandlungsoptionen chronischer Nierenerkrankungen haben sich in den zurückliegenden 10 Jahren deutlich verbessert und zwar in allen 3 Bereichen der Nierenerkrankungen: der präterminalen Niereninsuffizienz, dem Stadium der chronischen Dialysebehandlung und nach Nierentransplantation. Die betroffenen Patient:innen können daher trotz der Nierenerkrankung deutlich länger überleben.

Mit dem längeren Überleben geht die Notwendigkeit der kontinuierlichen medizinischen Versorgung und Betreuung einher und ebenso die Herausforderung, die damit verbundenen psychischen Belastungen zu bewältigen. Diese Herausforderung in medizinischer und psychologischer Sicht trifft alle Altersstufen der Patient:innen mit chronischen Nierenerkrankungen: sowohl Kinder und Jugendliche als auch Erwachsene bin ins Seniorenalter. In den Industrienationen sind die Senioren die am raschesten wachsende Gruppe von Patient:innen mit chronischer Nierenerkrankung, die aktuell mindestens 20 % der Dialysepopulation darstellt [1, 2].

Das Überleben mit Nierenerkrankung wird erschwert durch zahlreiche Komorbiditätsfaktoren und psychologische sowie soziale Probleme, mit denen die Patient:innen konfrontiert sind. Überleben ist nicht alles – daher muss die ganzheitliche Versorgung der Patient:innen mit chronischen Nierenerkrankungen verbessert werden. Konzeptionell werden Projekte im Rahmen der biopsychosozialen Klassifikation (ICF-Modelle; „international classification of functioning“) helfen, noch vorhandene Wissenslücken zu schließen und fehlende strukturelle Voraussetzungen neu zu schaffen [3–5].

In dieser kurzen Übersicht werden zunächst allgemeine Informationen zu Verbreitung, Ursachen und Therapiemöglichkeiten von chronischen Nierenerkrankungen gegeben. Im Anschluss werden die möglichen körperlichen und psychischen Langzeitfolgen im Zusammenhang mit der Erkrankung, den Begleiterkrankungen und der Therapie beschrieben. Auf bisher bekannte Auswirkungen der COVID-19-Pandemie wird eingegangen. Die Notwendigkeit einer multidisziplinären Langzeitversorgung von chronischen Nierenerkrankungen und weiterer Maßnahmen wird abschließend aufgezeigt.

## Chronische Nierenerkrankungen

Die Anzahl der Patient:innen mit chronischer Nierenerkrankung steigt global weiter an, auch wenn in den Industrienationen dank besserer medizinischer Versorgung der Anstieg etwas verlangsamt werden konnte. Global versterben nach Schätzungen 5 Mio. Patient:innen jährlich mit chronischen Nierenerkrankungen, da sie keinen Zugang zu einer entsprechenden medizinischen Versorgung haben. Bis 2040 könnten chronische Nierenerkrankungen die fünfthäufigste Todesursache weltweit sein [6, 7].

Das Erkennen und die frühzeitige Behandlung von Nierenerkrankungen bleibt eine Herausforderung, da sie meist lange unerkannt bleiben, häufig nicht rechtzeitig diagnostiziert werden und damit erst spät – meist zu spät – eine Therapie begonnen wird. Das frühzeitige Erkennen von Nierenerkrankungen und die rasche Zuweisung zu einer nephrologischen Behandlung sind jedoch erforderlich, um die Progression von Nierenerkrankungen zu verlangsamen [8–10]. Screeninguntersuchungen zur Prävention von Nierenerkrankungen haben sich bislang mangels sensitiver Marker nicht etablieren können. Entwicklungen in der personalisierten Medizin wie die Erstellung von Datenbanken, in denen Forschungsdaten und patientenbezogene Daten zusammengeführt werden, können in Zukunft dazu beitragen, Nierenerkrankungen frühzeitig zu erkennen und deren individuellen Verlauf besser vorherzusagen [11, 12].

Nierenerkrankungen („chronic kidney diseases“ – CKD) werden generell in 5 Stadien eingeteilt und verlaufen mit oder ohne Proteinurie. Die CKD-Stadien sind relevant für die Komplikationsprognose und werden zusammen mit den zugrunde liegenden Erkrankungen detailliert dargestellt in [10, 13]. Das Stadium 5 entspricht der terminalen Niereninsuffizienz und wird bei einer glomerulären Filtrationsrate (GFR) < 15 mL/min erreicht. Der Verlauf von Nierenerkrankungen variiert stark in Abhängigkeit von der Grunderkrankung und der zur Verfügung stehenden Therapie. Die Nierenfunktion kann sich, wie beim akuten Nierenversagen, weitgehend erholen, jedoch trotz aller therapeutischen Bemühungen auch rasch schlechter werden und innerhalb von wenigen Wochen zur terminalen Niereninsuffizienz führen, wie etwa bei rapid progressiven Glomerulonephritiden. Eine vollständige Ausheilung nach einer Nierenerkrankung ist prinzipiell nicht möglich, immer handelt es sich um eine Defektheilung, die mit einem Verlust an funktioneller Reserve einhergeht und damit das Risiko in sich trägt, im Intervall, häufig noch nach Jahren oder Jahrzehnten, zur terminalen Niereninsuffizienz zu führen. Ursächlich hierfür sind die Hyperperfusion und Hyperfiltration mit Albuminurie noch vorhandener und zunächst noch funktionstüchtiger Nephrone und deren nachfolgende Adaptationsprozesse [14]. Reparationsprozesse in der Niere anzustoßen, um eine vollständige Ausheilung zu erreichen, ist einer der großen aktuellen Forschungsschwerpunkte; ob und wann eine klinische Anwendung möglich sein wird, ist aktuell noch offen [15].

Mögliche Ursachen von Nierenerkrankungen sind vielfältig und reichen von 1) dem akuten Nierenversagen (z. B. im Rahmen einer Sepsis mit SIRS („systemic inflammatory response syndrome“) bei Intensivpatient:innen), 2) dem akut auf chronischen Nierenversagen (z. B. bei vorbekannter chronischer Niereninsuffizienz und passager auftretendem Durchfall mit Volumenverlust), 3) den eigenständigen Nierenerkrankungen (z. B. membranöse Glomerulonephritis), 4) den angeborenen Nierenerkrankungen (häufiger Beginn im Kindesalter, jedoch auch Spätmanifestation wie bei polyzystischen Organdegenerationen) bis hin zur 5) Nierenbeteiligung im Rahmen von System- und Autoimmunerkrankungen (z. B. Lupus erythematodes) und 6) der Nephrotoxizität von Medikamenten oder bei chronischem Schmerzmittel- und Drogenmissbrauch.

Zunehmende Bedeutung im Rahmen der chronischen Niereninsuffizienz gewinnt die Gruppe von Patient:innen, bei denen eine andere Grunderkrankung erfolgreich therapiert wurde, in deren Verlauf sich dann die chronische Niereninsuffizienz einstellt (z. B. nach erfolgreicher Chemotherapie einer malignen Grunderkrankung oder nach erfolgreicher Organtransplantation, wie z. B. nach Herz- oder Lebertransplantation).

Die medikamentöse Therapie von Nierenerkrankungen richtet sich nach der Grunderkrankung und reicht von der generellen Nephroprotektion mittels ACE-Hemmern oder AT1-Rezeptorantagonisten, der Gabe von Aldosteronantagonisten (selektive Mineralokortikoidrezeptor-Antagonisten) und von SGLT2(„sodium-dependent-glucose-co-transporter-2“)-Inhibitoren bis hin zur immunmodulierenden Therapie mit z. B. Cyclophosphamid, Rituximab, Mycophenolsäurederivaten, Calcineurinhemmern oder IL17(Interleukin-17)-Antagonisten.

Am häufigsten entstehen chronische Nierenerkrankungen infolge einer arteriellen Hypertonie, eines Diabetes mellitus oder einer Adipositas mit metabolischem Syndrom [16, 17]. Eine frühzeitige und moderne Therapie ist gerade bei dieser Patient:innengruppe möglich und kann erfolgreich sein, das Stadium der terminalen Niereninsuffizienz zu vermeiden. Voraussetzung ist allerdings, dass mit der intensivierten Therapie früh begonnen wird, die Therapieziele erreicht werden (Blutdruck 120:80 mm Hg und Stoffwechselführung HbA1c < 6,3 %) und die Patient:innen durch entsprechende Schulung, Gewichtsreduktion und regelmäßige sportliche Aktivität in die Therapie der Erkrankung eingebunden werden. Sie sollten zudem willens sein, die Therapieziele zu erreichen. Für eine detaillierte Darstellung der Therapieziele abhängig vom Stadium der Nierenerkrankung und Alter der Patient:innen sei an dieser Stelle auf die Leitlinien der Arbeitsgemeinschaft der Wissenschaftlichen Medizinischen Fachgesellschaften (AWMF) und KDIGO-Guidelines (Kidney Disease: Improving Global Outcomes) verwiesen [10, 13].

## Folgen chronischer Nierenerkrankungen

Die Konsequenzen des Verlustes der Nierenfunktion sind nicht auf die Niere als Organ limitiert, sondern haben Auswirkungen auf den Gesamtorganismus [18]. Diese Folgeschäden betreffen vor allem das Herz-Kreislauf-System, die Lunge, den Knochenstoffwechsel und das Knochenmark (Anämie, Infektanfälligkeit), den Gastrointestinaltrakt und das zentrale Nervensystem und entstehen durch die Kontamination des Blutes mit harnpflichtigen Substanzen (Urämie). Im Rahmen der Urämie können weitere nichtrenale Begleiterkrankungen bzw. -erscheinungen wie Depression, Schmerzsyndrom, Erschöpfung (Fatigue), Mangelernährung (Malnutrition) und Gebrechlichkeit (Frailty), Immobilität und Sturzneigung auftreten, die insbesondere in Bezug auf Lebensqualität und Mortalität bedeutsam sind. Die Urämie und ihre Folgeschäden treten nicht erst bei terminaler Niereninsuffizienz auf, sondern bereits bei einer nur moderat eingeschränkten Nierenfunktion. Zusätzliche Komplikationen können bei Nierenerkrankungen als Nebenwirkungen einer möglicherweise erforderlichen aggressiven Therapie, wie z. B. bei Autoimmunerkrankungen, auftreten.

Aufgrund dieser Multimorbidität müssen Patient:innen mit Nierenerkrankungen immer langfristig und multidisziplinär betreut werden. Zudem sollten die Patient:innen früh in die Therapieentscheidungen eingebunden werden. Sie müssen die Therapieziele kennen sowie motiviert und unterstützt werden, langfristig am Erreichen dieser Ziele mitzuwirken [19].

Dabei ist es wichtig, die mögliche psychische Belastung der Patient:innen und ihrer Angehörigen zu berücksichtigen, die sich auch aus dem Wissen um die multiplen, organübergreifenden somatischen Folgen, den möglichen Komplikationen, der erforderlichen langfristigen Betreuung und der Furcht vor einer weiteren Verschlechterung der Nierenfunktion ergeben kann [20–23].

## Folgen der Dialysebehandlung

Schreitet der Verlust an Nierenfunktion voran, ist der Beginn eines Nierenersatzverfahrens erforderlich. Prinzipiell möglich sind die Hämodialyse, die Peritonealdialyse und die Nierentransplantation (s. unten). Im Stadium der terminalen Niereninsuffizienz durchlaufen die Patient:innen häufig verschiedene Behandlungsverfahren, z. B. beginnend mit der Peritonealdialyse über eine Nierentransplantation bis hin zur Hämodialyse aufgrund des Verlustes der Transplantatfunktion.

Die Dialysetechnik hat sich seit ihren Anfängen durch die fundamentalen Arbeiten von Willem Kolff und Belding Scribner nachhaltig weiterentwickelt. Trotz aller Fortschritte in der Dialysetechnik bleibt die Mortalität der Dialyse-Patient:innen aber insgesamt inakzeptabel hoch. Mit Eintreten der terminalen Niereninsuffizienz steigt die Morbiditäts- und Mortalitätsrate der Patient:innen deutlich an, und zwar bei jüngeren Patient:innen um den Faktor 4–7, bei über 60-jährigen Patient:innen um das 20- bis 30-Fache im Vergleich zum gesunden Kontrollkollektiv gleichen Alters.

Nach aktueller Schätzung erhalten derzeit weltweit etwa 2,5 Mio. Menschen eine Dialysebehandlung, 2030 werden es voraussichtlich 5,4 Mio. sein. Das sind die realisierten Dialysen; der Bedarf an Dialysebehandlungen liegt weit höher bei etwa 10 Mio. Die Kosten für die Dialysebehandlung nehmen ca. 5–7 % des gesamten Gesundheitsbudgets in Anspruch, obwohl der Prozentsatz der betroffenen Patient:innen aktuell nur bei etwa 0,2 % der Gesamtbevölkerung liegt [24]. Mit einer Zunahme der Zahl dialysepflichtiger Patient:innen wird die Belastung des Gesundheitssystems weiter ansteigen. Diese Belastung ergibt sich bei der terminalen Niereninsuffizienz nicht ausschließlich aus den Dialysebehandlungskosten, sondern auch aus den Kosten für die erforderlichen Medikamente, Krankenhausaufenthalte und Transporte zur Dialysebehandlung. Die reinen Behandlungskosten der Hämodialysebehandlung liegen in Deutschland jährlich bei ca. 93.000 €, die der Peritonealdialyse bei ca. 78.000 € und die der Nierentransplantation bei etwa 37.000 €. Die häufig jüngeren Patient:innen werden vom Sozialsystem übernommen. Der Verlust des Arbeitsplatzes und die damit verbundene soziale Isolation sind für viele der Patient:innen ein erhebliches Problem – und für die Gemeinschaft entstehen zusätzliche Kosten [24, 25].

Die Dialyse verhindert den unmittelbaren Tod durch das Nierenversagen, geht jedoch mit einer deutlich verkürzten Lebenserwartung, einer hohen Hospitalisierungsrate und einem deutlichen Verlust an Lebensqualität einher. Leider erreichen viele, auch jüngere Patient:innen das Stadium der terminalen Niereninsuffizienz unvorbereitet sowohl unter medizinischen wie auch unter medizinpsychologischen Aspekten. Bei sehr alten und betagten, meist auch multimorbiden Patient:innen sollte die Entscheidung, mit der Dialysebehandlung zu beginnen oder die konservative Therapie bis zum Lebensende fortzuführen, sehr sorgfältig abgewogen werden [26–28].

Die Hämodialysebehandlung erfolgt i. d. R. dreimal pro Woche für die Dauer von 4–5 h und kann bei ausgewählten Patient:innen auch als Heimhämodialysebehandlung durchgeführt werden. Als Alternative steht die Peritonealdialysebehandlung zur Verfügung, die Patient:innen weitgehend eigenständig und kontinuierlich an 7 Tagen in der Woche durchführen (auch eine nächtliche Peritonealdialyse ist möglich).

Die für die Patient:innen wahrscheinlich beste Form der Dialysebehandlung ist die heimbasierte Dialyse als Hämodialyse oder Peritonealdialyse, unterstützt durch telemedizinische Betreuung der Patient:innen im angeschlossenen Dialysezentrum. Die heimbasierte Dialyse trägt zur besseren Lebensqualität, geringeren Mortalität und Morbidität der Patient:innen bei [29–31].

Das Stadium der terminalen Niereninsuffizienz geht für die Patient:innen mit weiteren Belastungen einher. Diese betreffen medizinische Komplikationen (z. B. rasch fortschreitende Gefäßsklerose und koronare Herzerkrankung), die Einnahme zusätzlicher Medikamente, Einschränkungen bezüglich Ernährung und Trinkmenge sowie zeitliche Einschränkungen durch die Transportzeiten, die Dauer der Dialysebehandlung sowie die nötigen Erholungsphasen nach den Dialysebehandlungen – um nur die wichtigsten Aspekte zu nennen.

Mit einer terminalen Niereninsuffizienz sind häufig immense psychische Belastungen verbunden, die sich aus dem (drohenden) Verlust der sozialen Integration oder des Ausbildungs- oder Arbeitsplatzes, aber auch aus den Begleiterkrankungen bzw. -erscheinungen wie Depressionen, Schlafstörungen, neurokognitive Einschränkungen und Fatiguesyndrom ergeben können. Bei Kindern kann sich eine Wachstumsretardierung belastend auswirken. Familiäre Beziehungen können gefährdet sein [19, 20, 32–39]. Die medizinpsychologischen Folgen sind altersspezifisch und müssen im Rahmen der Transition von der Kinder- in die Erwachsenennephrologie und bei der Behandlung älterer Patient:innen Berücksichtigung finden [40–42].

### Ganzheitliche Versorgung von Dialysepatient:innen

Dialyse alleine ist nicht ausreichend! Die Patient:innen müssen immer ganzheitlich behandelt werden. Das bedeutet, dass neben der medizinischen Versorgung immer auch Psychotherapie und begleitende psychosoziale Betreuung angeboten werden sollten sowie Unterstützung und Beratung in Hinblick auf eine gesunde Lebensführung (Lifestyle Interventions; [43–48]). Die ganzheitliche Versorgung der Patient:innen mit CKD umfasst die Versorgung unter allen Aspekten (Total Renal Care; [49]).

Die Patient:innen sollten in ihrem Selbstmanagement unterstützt und in alle therapeutischen Entscheidungen eingebunden werden (Shared Decision Making; [50–54]). Sie müssen die Tragweite der Entscheidungen verstehen und diese mitverantworten können.

Ein weiterer Faktor, der bei der ganzheitlichen Versorgung beachtet werden muss, ist, dass sich Patient:innen zwar mit zunehmender Dauer der Dialysebehandlung an die erforderlichen Einschränkungen gewöhnen (Adaptation an den Stressor), Veränderungen in der gewohnten Dialyseumgebung, wie neues Personal, neue Maschinen oder Zu- und Abgang von Mitpatient:innen aber Angst und Verunsicherung auslösen können.

Leider fehlen vielerorts die strukturellen und finanziellen Voraussetzungen zur optimalen und ganzheitlichen Versorgung und Betreuung der dialysepflichtigen Patient:innen. Weitere Informationen zu diesem Thema stellt die European Kidney Health Alliance zur Verfügung (http://ekha.eu/).

## Folgen der Nierentransplantation

Sowohl im Hinblick auf Lebensqualität und Überleben als auch unter gesundheitsökonomischen Gesichtspunkten ist die Nierentransplantation aktuell die beste Form der Versorgung von Patient:innen mit terminaler Niereninsuffizienz. Aufgrund der Multimorbidität der Dialysepatient:innen kann die Nierentransplantation jedoch nicht allen Patient:innen angeboten werden. Wegen des weiterbestehenden eklatanten Organmangels und der damit verbundenen langen Wartezeiten bis zur Nierentransplantation (aktuell etwa 10 Jahre) können einige Patient:innen, obwohl primär geeignet für die Nierentransplantation, u. U. nach Ablauf der Wartezeit nicht mehr zur Transplantation akzeptiert werden [55–57].

Mit der langen Wartezeit auf ein passendes Spenderorgan sind nicht nur die medizinischen Probleme verbunden, sondern immer auch Ängste, wie etwa die Sorge, dass ein Organangebot doch bald eintrifft, oder Bedenken, ob die Transplantation erfolgreich verlaufen wird und das transplantierte Organ auch möglichst lange funktionstüchtig bleibt [58].

Nach der Transplantation benötigen die Patient:innen weiterhin engmaschige und regelmäßige Kontrolluntersuchungen. Vorhandene zusätzliche medizinische Probleme (z. B. eine koronare Herzerkrankung) bleiben bestehen, auch wenn eine Verzögerung des Fortschreitens der Begleiterkrankungen nach Nierentransplantation eintritt. Außerdem können die Nebenwirkungen, die durch die erforderliche immunsuppressive Therapie auftreten, gravierend sein [59–61].

Die 1‑Jahres-Funktionsrate von Transplantaten hat sich in den zurückliegenden 3 Jahrzehnten nachhaltig verbessert, leider sind wir von dem Ziel, die Patient:innen nur einmal transplantieren zu müssen und dann die Transplantatfunktion auf Dauer erhalten zu können, noch weit entfernt [62]. Auch wenn intensiv an einer Verbesserung des Langzeiterfolges nach Transplantation gearbeitet wird (*Stichworte* Biomarker, Telemedizin, Toleranzinduktion, Maschinenperfusion der Organe vor Transplantation), bleiben Adhärenz und Mitarbeit der Patient:innen zentraler Bestandteil des Erfolges nach Nierentransplantation. Mindestens 50 % der Organe gehen aktuell frühzeitig aufgrund mangelnder Adhärenz der Patient:innen verloren [63, 64]. Studien zur Verbesserung der Adhärenz von Patient:innen nach Nierentransplantation werden als Teil von Langzeitinterventionsuntersuchungen durchgeführt; erste Ergebnisse sind bereits publiziert [65–67].

Idealerweise findet eine Nierentransplantation vor Beginn einer nötig werdenden Dialysebehandlung statt, wobei das Transplantat von einem lebenden Organspender stammt (präemptive Lebendspendentransplantation). Diese kann leider nicht allen Patient:innen angeboten werden [68]. Die Spender müssen medizinisch gesund sein (und dies, soweit Vorhersagen möglich sind, auch bleiben), sich zudem medizinpsychologisch für die Nierenspende qualifizieren und den mit der Spende einhergehenden Belastungen gewachsen sein. Die Spender benötigen nach der Nierenspende medizinische Verlaufskontrollen (u. a. Nierenfunktion, Blutdruck, Diabetes) und medizinpsychologische Nachsorgeuntersuchungen, um frühzeitig Belastungssituationen (Fatigue, Depression) erkennen und ggf. behandeln zu können.

Sind die – meist jüngeren – Patient:innen erfolgreich transplantiert und liegt die Transplantation lange zurück, dann erreicht die medizinische und psychologische Rehabilitation der Patient:innen fast das Niveau des Kontrollkollektives gleichen Alters [69].

## COVID-19-Pandemie und Niereninsuffizienz

Die COVID-19-Pandemie hat nachdrücklich verdeutlicht, wie vulnerabel die Gruppe der Patient:innen mit chronischen Nierenerkrankungen ist. Auch wenn es noch Jahre dauern wird, alle Aspekte der COVID-19-Erkrankung zu untersuchen und zu verstehen, die Einfluss auf den Verlauf von Nierenerkrankungen, die Dialyse und die Nierentransplantation haben, ist bereits jetzt klar, dass Patient:innen mit einer chronischen Nierenerkrankung nach einer SARS-CoV-2-Infektion eine schlechtere Prognose haben [70, 71]. Und umgekehrt haben Patient:innen mit einem schweren Verlauf der COVID-19-Erkrankung ein erhöhtes Risiko, ein akutes Nierenversagen und im weiteren Verlauf eine chronische Niereninsuffizienz zu entwickeln [72–75]. Die Diskussionen zur besten Form der Therapie des akuten Nierenversagens bei Patient:innen mit schwerer intensivpflichtiger COVID-19 sind noch nicht abgeschlossen [76].

Während der COVID-19-Pandemie ist die Zahl der Nierentransplantationen weltweit deutlich zurückgegangen [77, 78]. Die Behandlung von bereits transplantierten Patient:innen unter immunsuppressiver Medikation, die an COVID-19 erkranken, ist schwierig – sowohl im Hinblick auf die Modifikation der immunsuppressiven Therapie zum Erhalt der Transplantatfunktion wie auch im Hinblick auf die optimale Therapie von COVID-19 [77, 79–81].

Welche Langzeitfolgen eine schwere COVID-19-Erkrankung mit Nierenbeteiligung auf die Inzidenz von Nierenerkrankungen und ggf. die Anzahl dialysepflichtiger Patient:innen insbesondere nach schwerer COVID-19-Erkrankung im Kindesalter haben wird, ist bislang unbekannt und wird weiter beobachtet und aufgearbeitet werden müssen [82].

Impferfolge mit den aktuell zur Verfügung stehenden COVID-19-Impfstoffen sind bei Patient:innen mit chronischen Nierenerkrankungen, insbesondere an Dialyse und nach Nierentransplantation deutlich seltener zu verzeichnen als bei der allgemeinen Bevölkerung gleichen Alters. Impfstrategien müssen daher gerade bei diesem Patient:innenkollektiv weiter verbessert werden [83–86].

Nicht nur die medizinischen Folgen einer COVID-19-Erkrankung hinsichtlich des Verlaufs einer chronischen Nierenerkrankung sind bislang unzureichend bekannt, auch die medizinpsychologischen Folgen für dieses kritische Patient:innenkollektiv sind noch weitgehend unerforscht und müssen in den kommenden Jahren aufgearbeitet werden [87, 88]. Ebenfalls zu untersuchen sind die medizinpsychologischen Folgen der Pandemie bei Patient:innen mit dialysepflichtiger Niereninsuffizienz, die auf der Warteliste zur Nierentransplantation stehen und unsicher sind, wann ein Organangebot sie erreichen wird und ob sie das Organangebot dann annehmen oder mit Blick auf eine mögliche SARS-CoV-2-Infektion eher auf einen späteren Zeitpunkt und ein erneutes Organangebot verschieben sollten [89, 90].

Die Auswirkungen einer Long-COVID-Erkrankung bei Patient:innen mit chronischen Nierenerkrankungen und nach Nierentransplantation wurden bisher nicht untersucht und es wird erheblicher Anstrengungen bedürfen, Long-COVID besser zu verstehen [91–96].

Die COVID-19-Pandemie hat noch einen allgemeinen Aspekt der medizinischen Versorgung in das Blickfeld der Öffentlichkeit gerückt: die Priorisierung bei der Ressourcenverteilung in Zeiten der Ressourcenknappheit. Nicht nur im Rahmen von Pandemien, sondern auch im Hinblick auf eine älter werdende Gesellschaft und immer teurer werdende Therapiemöglichkeiten muss dieses Thema intensiv diskutiert und weiter um Lösungen für schwierige ethische Entscheidungen gerungen werden [97–101].

## Ausblick

**Figure 1:**
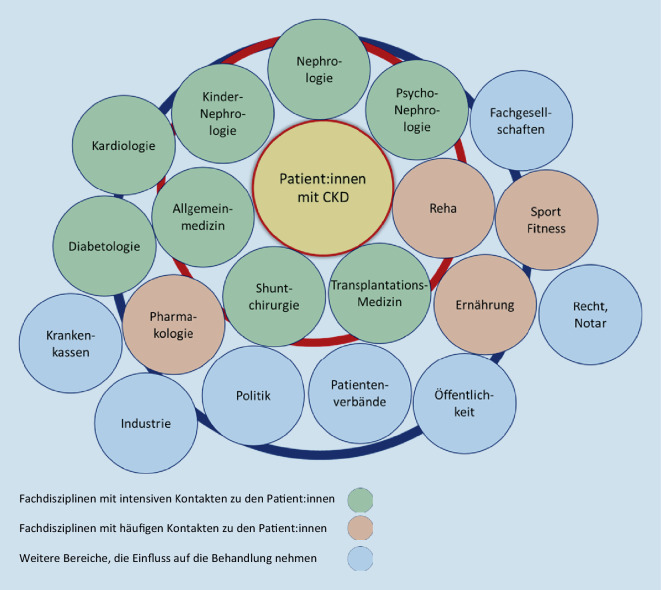

Die Langzeitversorgung von Patient:innen mit chronischen Nierenerkrankungen, Dialyse und nach Nierentransplantation ist bei allen Fortschritten weiterhin defizitär. Die Patient:innen benötigen eine frühzeitige und oft über Jahrzehnte andauernde kontinuierliche multidisziplinäre Versorgung durch Fachärzt:innen für (Kinder‑)Nephrologie und Psychonephrologie unter Einbeziehung von Allgemeinmedizin, Diabetologie, Kardiologie, Shuntchirurgie, Transplantationschirurgie, Pharmakologie, Ernährungsberatung, Fitnessberatung und Rehabilitationskliniken sowie bei Bedarf weiterer Disziplinen ([102]; Abb. 1).

Die Integration der Medizin und Psychologie während der ambulanten und stationären Betreuung der Patient:innen muss gewährleistet sein, es sollte in der Betreuung der komplex erkrankten Patient:innen keine Unterbrechungen geben (Abb. 2). Barrieren zwischen den verschiedenen Behandlungsebenen müssen im Rahmen eines Konsensusprozesses wissenschaftlich begründet und Möglichkeiten zu deren Überwindung erarbeitet werden und zusätzlich müssen epidemiologische Aspekte, verfügbare Personalressourcen sowie Kosten und Strukturen des Gesundheitssystems berücksichtigt werden [11]. Tele-Health, Telenephrologie und Telepsychologie können helfen, die Situation graduell zu verbessern [103, 104]. Aber es bedarf fester organisatorischer, struktureller und finanzieller Voraussetzungen zur optimalen Betreuung der Patient:innen mit chronischer Nierenerkrankung [105, 106]. Die komplexe Betreuung der betroffenen Patient:innen kann auf Dauer nicht dem individuellen Engagement der ärztlichen und pflegerischen Kolleg:innen überlassen bleiben [107].

**Figure 2:**